# The Prevalence of Electrocardiogram (ECG) Changes in Patients on Clozapine

**DOI:** 10.1192/bjo.2022.446

**Published:** 2022-06-20

**Authors:** Rachael Howson, Zain Rana, Micheal Kurkar

**Affiliations:** Pennine Care NHS Foundation Trust, Oldham, United Kingdom

## Abstract

**Aims:**

Clozapine is an atypical antipsychotic primarily used in the management of individuals with schizophrenia and schizoaffective disorders, prescribed to those with symptoms unresponsive to alternate antipsychotic medications. Clozapine is known to have cardiovascular side effects and is associated with an increased risk of significant cardiac events including myocarditis, cardiomyopathy, and sudden cardiac death. Regular electrocardiogram (ECG) monitoring is recommended to facilitate early detection of cardiac complications. This study aimed to identify the prevalence and evaluate the nature of ECG changes, assessing for tachycardia and corrected QT (QTc) interval prolongation, in patients prescribed Clozapine, and to determine whether the appropriate action was taken following identification of these changes.

**Methods:**

We conducted retrospective data collection examining consecutive ECGs of 50 adult patients prescribed Clozapine within the East sector of the Cherrywood Outpatient Psychiatry Department at The Royal Oldham Hospital. Patients were identified using the clinic's Clozapine database. The PARIS electronic record system and patient written notes were utilised to obtain patient demographics, diagnoses, and ECGs. We assessed rate, rhythm and QTc intervals amongst the ECGs taken and compared the most recent ECG findings with those from previous ECGs.

**Results:**

Of the 50 patients prescribed Clozapine, 34 were identified as having 2 consecutive ECGs in their notes, enabling ECG comparison and assessment for changes. Amongst these 34 patients, 11 (32.35%) demonstrated new-onset ECG changes; 8 with new sinus tachycardia, 1 with new QTc prolongation and 2 with additional rhythm strip abnormalities. Based on these new findings, 50% were then referred for a repeat ECG. No plan had been made for the other 50%. ECGs of 8 (23.53%) individuals demonstrated changes which remained present across the consecutive ECGs. Plans for these patients included referral for cardiology opinion (25%), repeat ECG (25%) and dose reduction (50%). A further 8 patients demonstrated an improvement in findings on their most recent ECG. In 3 (37.50%) of these cases, Clozapine had been reduced during the period between ECG recordings. 7 (20.59%) individuals demonstrated no ECG changes.

**Conclusion:**

Our findings suggest many individuals prescribed Clozapine develop ECG abnormalities, with the largest proportion developing sinus tachycardia. Regular monitoring remains beneficial within the outpatient department to determine the nature of ECG changes, and further methods may be required to ensure appropriate management plans are in place should these changes arise.

